# The long-term effectiveness of cognitive behavior therapy for psychosis within a routine psychological therapies service

**DOI:** 10.3389/fpsyg.2015.01658

**Published:** 2015-10-29

**Authors:** Emmanuelle Peters, Tessa Crombie, Deborah Agbedjro, Louise C. Johns, Daniel Stahl, Kathryn Greenwood, Nadine Keen, Juliana Onwumere, Elaine Hunter, Laura Smith, Elizabeth Kuipers

**Affiliations:** ^1^Department of Psychology, Institute of Psychiatry, Psychology and Neuroscience, King’s College LondonLondon, UK; ^2^Psychological Interventions Clinic for Outpatients with Psychosis, South London and Maudsley NHS TrustLondon, UK; ^3^NIHR Biomedical Research Centre for Mental Health, South London and Maudsley NHS Trust, King’s College LondonLondon, UK; ^4^Department of Biostatistics, Institute of Psychiatry, Psychology and Neuroscience, King’s College LondonLondon, UK; ^5^Department of Psychology, Sussex UniversitySussex, UK; ^6^Sussex Partnership NHS Foundation TrustSussex, UK

**Keywords:** cognitive behaviour therapy, psychosis, schizophrenia, effectiveness, randomised controlled trials

## Abstract

Randomised controlled trials (RCTs) have shown the efficacy of CBTp, however, few studies have considered its long-term effectiveness in routine services. This study reports the outcomes of clients seen in a psychological therapies clinic, set up following positive results obtained from an RCT (Peters et al., 2010). The aims were to evaluate the effectiveness of CBTp, using data from the service’s routine assessments for consecutive referrals over a 12 years period, and assess whether gains were maintained at a 6+ months’ follow-up. Of the 476 consenting referrals, all clients (*N* = 358) who received ≥5 therapy sessions were offered an assessment at four time points (baseline, pre-, mid-, and end of therapy) on measures assessing current psychosis symptoms, emotional problems, general well-being and life satisfaction. A sub-set (*N* = 113) was assessed at a median of 12 months after finishing therapy. Following the waiting list (median of 3 months) clients received individualized, formulation-based CBTp for a median number of 19 sessions from 121 therapists with a range of experience receiving regular supervision. Clients showed no meaningful change on any measure while on the waiting list (Cohen’s *d* <= 0.23). In contrast, highly significant improvements following therapy, all of which were significantly greater than changes during the waiting list, were found on all domains assessed (Cohen’s d: 0.44–0.75). All gains were maintained at follow-up (Cohen’s d: 0.29–0.82), with little change between end of therapy and follow-up (Cohen’s *d* <= 0.18). Drop-out rate from therapy was low (13%). These results demonstrate the positive and potentially enduring impact of psychological therapy on a range of meaningful outcomes for clients with psychosis. The follow-up assessments were conducted on only a sub-set, which may not generalize to the full sample. Nevertheless this study is the largest of its kind in psychosis, and has important implications for the practice of CBTp in clinical services.

## Introduction

Cognitive behavior therapy (CBT) for psychosis (CBTp) is an adaptation of CBT for emotional disorders, tailored to the specific needs of people with psychosis. The heterogeneity of presentation in psychosis means that therapy approaches are diverse, with up to 30 books and manuals currently available [see (Johns et al., 2014) for a full list]. Broadly, the aims of CBTp are to work collaboratively with the person to help them gain a better understanding of their psychotic experiences and potential contributing factors; enhance coping and improve functioning; learn adaptive strategies to manage emotional distress; break vicious cycles by identifying cognitive processes and behaviors that are maintaining the problem; and consider alternative, less distressing ways of appraising their experiences. The main instrument of change in CBTp involves making changes in appraisals and behavior to reduce distress, in the context of a good therapeutic relationship.

There is now a robust evidence base demonstrating that CBTp can produce improvements in a variety of outcomes in patients who continue to have residual psychosis symptoms and emotional difficulties despite optimal medication. This body of work has led to its current status as a recommended treatment within the UK National Institute for Health and Clinical Excellence (NICE, 2014), American Patient Outcomes Research Team (PORT; (Kreyenbuhl et al., 2010), and international (Gaebel et al., 2011) guidelines for psychosis and schizophrenia. To date, there have been 12 meta-analyses reviewing up to 50 randomised controlled trials (RCTs), including five within the last year (Burns et al., 2014; Jauhar et al., 2014; Turner et al., 2014; van der Gaag et al., 2014; Velthorst et al., 2015). The effect sizes across the different meta-analyses are small to moderate, ranging from 0.09 to 0.49, depending on trials included and outcomes examined. Two of the larger meta-analyses reported an inverse relationship between effect size and methodological rigor, especially blinding (Wykes et al., 2008; Jauhar et al., 2014), suggesting caution in interpreting previous positive outcomes of CBTp. However, the value of combining highly heterogeneous trials with different foci has been questioned (Byrne, 2014; Peters, 2014), since such analyses reflect an over-simplification of the complexities of psychosis presentations and of the range of psychological interventions encompassed within a broad CBTp framework. Other recent meta-analyses, which focus on treatment-resistant patients [effect size: 0.47; (Burns et al., 2014)], or on individually tailored, formulation-based CBT for hallucinations (effect size: 0.44) and delusions [effect size: 0.36; (van der Gaag et al., 2014)] are more informative about the specific effects of CBTp.

The focus on symptom severity as a primary outcome has also been criticized, since CBTp targets symptom distress and impact on functioning, as well as psychological recovery, rather than symptom reduction *per se* (Birchwood and Trower, 2006). Trials that have used ‘psychological’ outcomes rather than symptom scores, such as compliance with command hallucinations (Trower et al., 2004), global functioning (Grant et al., 2012), or psychological well-being (Freeman et al., 2014), have tended to report higher effect sizes. Recent research has focused on targeted therapies that evaluate individual components of therapy focusing on specific processes, such as worry (Freeman et al., 2015) or reasoning biases (Waller et al., 2011; Moritz et al., 2014; Garety et al., 2015), or specific sub-populations such as psychosis individuals presenting with command hallucinations (Birchwood et al., 2014) or post-traumatic stress disorder (van den Berg et al., 2015).

However, in clinical practice therapy tends to cover a range of difficulties within the same individuals, including distressing psychotic experiences, emotional problems, and quality of life. In the UK, (NICE, 2014) recommend that therapy should be formulation-based, and delivered on an individual basis for at least 16 sessions over a period of six or more months. While there are a number of obstacles in implementing NICE guidance in practice (Berry and Haddock, 2008; Haddock et al., 2014), nevertheless mental health services across the UK are attempting to deliver CBTp routinely. To date, only a handful of studies have evaluated CBTp delivered in routine clinical services, mostly reporting effectiveness RCTs (Farhall et al., 2009; Peters et al., 2010; Lincoln et al., 2012). While RCTs are clearly the gold standard in informing evidenced-based practice, there are limitations that need to be considered when inferring their efficacy to real life clinical settings (Morrison et al., 2004). RCTs, even those conducted as effectiveness trials within routine services, often have certain characteristics, such as strict exclusion criteria and pre-defined primary outcomes. Furthermore, in routine services there is often a greater emphasis on the goals of the individual client, causing variation in the focus of therapy (Farhall et al., 2009). Therapists may differ in experience, profession and training levels, and there are often limitations on time and resources.

There is a rich literature in other mental health disorders on the need for ‘practice-based evidence,’ which contributes in its own right to the evidence base for the effectiveness of psychological therapies (Lucock et al., 2003; Stiles et al., 2008). For instance, (Ehlers et al., 2013) demonstrated that clinicians’ concerns that the good outcomes in efficacy trials of CBT for Post-Traumatic Stress Disorder (PTSD) would not generalize to the wider range of traumas and presentations seen in routine practice were not founded, with large improvements in PTSD symptoms being observed in a consecutive sample attending a psychological therapies clinic, and few of the selection criteria used in RCTS moderating treatment outcome. In contrast, (Quarmby et al., 2007) found that the outcomes for CBT for chronic fatig syndrome in a routine service were inferior to those found in a previous RCT, which the authors suggest may have stemmed from patient selection, therapist factors and the use of a manualised protocol in the RCT.

In psychosis the few naturalistic studies that have been carried out have been highly promising (Thomas et al., 2011; Jolley et al., 2015), although they have suffered from small sample sizes. In a slightly larger study (*N* = 57; Morrison et al., 2004) evaluated CBTp using non-expert therapists within a community mental health team (CMHT) setting. CBTp produced significant improvements in positive symptoms, general mental health problems, and depression, most of which were maintained at a 1-year follow-up. In the current study, we sought to extend this work by investigating the effectiveness of CBTp on a range of outcomes in a large, unselected consecutive sample attending a psychological therapies service. (Peters et al., 2010) previously reported positive outcomes on a number of variables in an effectiveness RCT conducted at the Psychological Interventions Clinic for outpatients with Psychosis (PICuP), based at the South London and Maudsley NHS Foundation Trust (SLaM), some of which were maintained at follow-up. The current study reports the outcomes of those patients seen in the clinical service, developed from the trial, using data collected over a 12 years period as part of the service’s routine assessments immediately post therapy and at a minimum of 6 months’ follow-up.

## Materials and Methods

### Service Setting

These data were collected at the Psychological Interventions Clinic for outpatients with Psychosis (PICuP), part of the Recovery Pathway of the SLaM Psychosis Clinical Academic Group (CAG), based in South London at the Maudsley Hospital, between 2003 and 2015. SLaM serves four London boroughs, each with high rates of diversity [50–60% Black and Minority Ethnic (BME) groups, Office for National Statistics, 2012], population movement, drug use, crime, socio-economic deprivation, and psychosis incidence. A minority of patients (<10%) were referred from other London and environs boroughs. PICuP is a stand-alone psychological therapies clinic offering CBTp for outpatients with distressing positive symptoms of psychosis, or with emotional difficulties in the context of a history of psychosis. Therapists liaise closely with care-coordinators in recovery multidisciplinary teams, but are not part of the team, and do not prescribe medication or care-coordinate/case-manage. PICuP was set-up as a National Health Service (NHS) clinic on the back of initial funding for the RCT (Peters et al., 2010), and has been running for 12 years.

### Participants

The PICuP database provided an initial sample of 510 consecutive referrals whose therapy was completed and/or had been discharged and/or whose follow-up period had elapsed. Thirty-four people were excluded because they did not give consent for their measures to be used for service evaluation purposes, leaving 476 participants. The remaining sample consisted of 266 (56%) men and 210 (44%) women, with a mean age of 39 years (*SD* = 9.9). Almost half of the clients were from BME groups (*N* = 205; 48%), and a substantial majority were single (*N* = 340; 76%). Of the sample, 237 (50%) presented with current auditory hallucinations, and 296 (62%) with delusions. 35% were in the severe range for depression [>28 on the Beck Depression Inventory-II; BDI-II (Beck et al., 1996)], and 38% for anxiety [>25 on the Beck Anxiety Inventory (BAI; Beck et al., 1988)]^1^.

### Measures

The assessments consisted of a battery of measures assessing current symptoms of psychosis, emotional problems, and quality of life. The choice of routine outcome measures selected by the service is reflective of the wide range of problems held by many clients attending PICuP, and the individualized nature of therapy and people’s goals (Peters et al., 2010). The PSYRATs scales (Haddock et al., 1999) were only administered to those clients presenting with hallucinations (*N* = 237) and delusions (*N* = 296). Pragmatic considerations typical of routine clinical services, such as financial constraints or Trust-wide initiatives, led to the discontinuation of some measures after a number of years (Beck Depression and Anxiety Inventories (Beck and Steer, 1990; Beck et al., 1996); Manchester Short Assessment of Quality of Life Questionnaire (MANSA; Priebe et al., 1999), and the introduction of others [Clinical Outcomes in Routine Evaluation-10 (CORE-10)] (Barkham et al., 2013).

#### Psychotic Symptom Rating Scales – Auditory Hallucinations (PSYRATS-H; Haddock et al., 1999)

Eleven item semi-structured interview including frequency, duration, location, loudness, beliefs about origin, negative content, distress, disruption to life and control. Each symptom is rated on a five-point ordinal scale (0–4) by the interviewer, and the total scores range from 0–44.

#### Psychotic Symptom Rating Scales – Delusions (PSYRATS-D; Haddock et al., 1999)

Six item semi-structured interview including preoccupation, conviction, distress, and disruption to life. Each symptom is rated on a five-point ordinal scale (0–4) by the interviewer, and the total scores range from 0–24.

#### Beck Depression (BDI-II; Beck et al., 1996) and Anxiety (BAI; Beck and Steer, 1990) Inventories

Twenty-one item self-report questionnaires assessing symptoms of depression and anxiety, respectively, over the past week (possible range 0–63).

#### Manchester Short Assessment of Quality of Life Questionnaire (MANSA; Priebe et al., 1999)

Twelve item self-report questionnaire assessing satisfaction with life as a whole and across various domains such as finances, leisure, and mental health (possible range 0–84).

#### Clinical Outcomes in Routine Evaluation-10 (CORE-10; Barkham et al., 2013)

Ten-item self-report questionnaire assessing emotional well-being. The CORE-10 generates a total distress score, based on each item being rated from 0 to 4, with total scores ranging from 0 (low) to 40 (severe).

### Therapy

All clients were offered approximately 6–9 months of therapy, although there was considerable variation across individuals in actual length of therapy received (median = 9; range = 3–34^2^; mode = 6). Overall 93% of the sample was seen for therapy within an 18-month therapy window.

The median number of therapy sessions attended was 19 (mode = 26, range = 5–63). Number of sessions was highly skewed, with only 13% receiving more than 26 sessions. While clients were in therapy with PICuP they continued to receive routine mental health care from their recovery team (such as medication and appointments with care-coordinators), or their General Practitioner (GP) if they had been discharged from their team, but they did not receive other psychosocial interventions.

Therapy was usually delivered in weekly or fortnightly 1-hour sessions, although again length of session was variable across clients. All of the therapists (*n* = 121) had received training in CBT but most were not experts in CBTp specifically. In addition to permanent staff and their clinical psychology trainees, a large number of therapists were employed in other roles by their NHS trust (e.g., clinical psychologist, psychiatrist, or nurse), and conducted the therapy during their Continuous Professional Development (CPD) sessions to develop their skills in CBTp. All therapists attended fortnightly group supervision sessions with a permanent senior member of staff and had access to a ‘therapy pack’ and a variety of reading materials. Clinical psychology trainees received individual weekly supervision, including listening to therapy tapes.

Therapy was conducted in a flexible style with an emphasis on engagement and building a good therapeutic relationship. Interventions were formulation-based and focused on the patient’s own goals, which, in addition to managing and understanding distressing positive symptoms, often centered on emotional problems and/or social inclusion (see Fowler et al., 1995; Johns et al., 2014).

### Procedure

Participants were assessed at four different time points as part of the routine outcome assessments for the clinic:

-  Baseline (when first referred to the service, before going on the waiting list);
-  Pre-therapy (just before starting therapy after having been on the waiting list median of 3 months after the baseline assessment (range 1–17 months; mode = 2);
-  Mid-therapy (median of 4 months after the second or baseline assessment (range 2–15 months; mode = 4);
-  Post-therapy (within a few days or weeks of finishing therapy; median of 5 months after the mid-therapy assessment (range 1–25 months; mode = 5);

There were two exceptions to this: clients did not complete the second assessment (pre-therapy) if the waiting list was <=2 weeks, and the MANSA (Priebe et al., 1999) was only administered at baseline and end of therapy assessments; both to minimize client burden.

Seven years following the start of the service, a fifth assessment time-point was added:

-  Follow-up (at a minimum of 6 months post therapy; median 12 months following end of therapy assessment, range 6–46, mode = 6).

It was attempted to follow up early clients when the follow-up assessments started to be implemented routinely, but only a small percentage could be located; as a result the data-set for these assessments is smaller than for the other time-points.

Outcomes at the mid-therapy assessment are not reported here, but were included in the repeated measurements model in order to further reduce potential bias created by missing values (see statistical analysis section below).

Independent assessors (assistant psychologists trained in administering all the measures) conducted the assessments. Assessments lasted between 45 and 90 min, and could be conducted over more than one session if necessary. Demographic information from participants was collected at the baseline assessment and from the standard ‘Patient Registration Form’ used by SLaM.

### Statistical Analysis

The software packages STATA (version 11.2) and SPSS (version 21) were used to run the statistical analyses using a two-sided 1% significance level^3^.

The effectiveness of CBTp was tested by the following comparisons:

-  Baseline vs. pre-therapy, to check stability of symptoms while on the waiting list
-  Pre-therapy vs. post-therapy, to assess change over the course of therapy
-  Change during waiting list (pre therapy – baseline) vs. during therapy (post therapy – pre-therapy), to test whether change was greater in the latter period than in the former
-  Pre-therapy vs. follow-up, to assess whether any changes were maintained +6 months following the end of therapy
-  Post-therapy vs. follow-up, to check stability between end of therapy and follow-up.

Longitudinal data were analyzed through repeated measurement models (mixed effects regression) by an independent statistician (DA). For each outcome a linear mixed model was run to compare the measurements at the five time points (baseline; pre-therapy; mid-therapy; end of therapy; and 6+ months follow-up), including all available data at each time point.

The model, called covariance pattern model (Brown and Prescott, 1996), analyses the repeated measurements nested within individuals, using an unstructured covariance matrix (which allows unequal variances and covariances between the different time points measures), under the missing data assumption of missing at random (MAR, which does not depend on the missing values being conditional on the observed data).

In order to assess the five comparisons listed above, contrasts were formally expressed and estimated using STATA’s ‘lincom’ function, and effects sizes (Cohen’s *d*) for the changes of interest were subsequently computed. Cohen’s *d* was calculated by dividing the absolute mean change estimate by the standard deviation of the mean baseline measure.

## Results

### Therapy and Assessment Attrition

**FIGURE 1:**
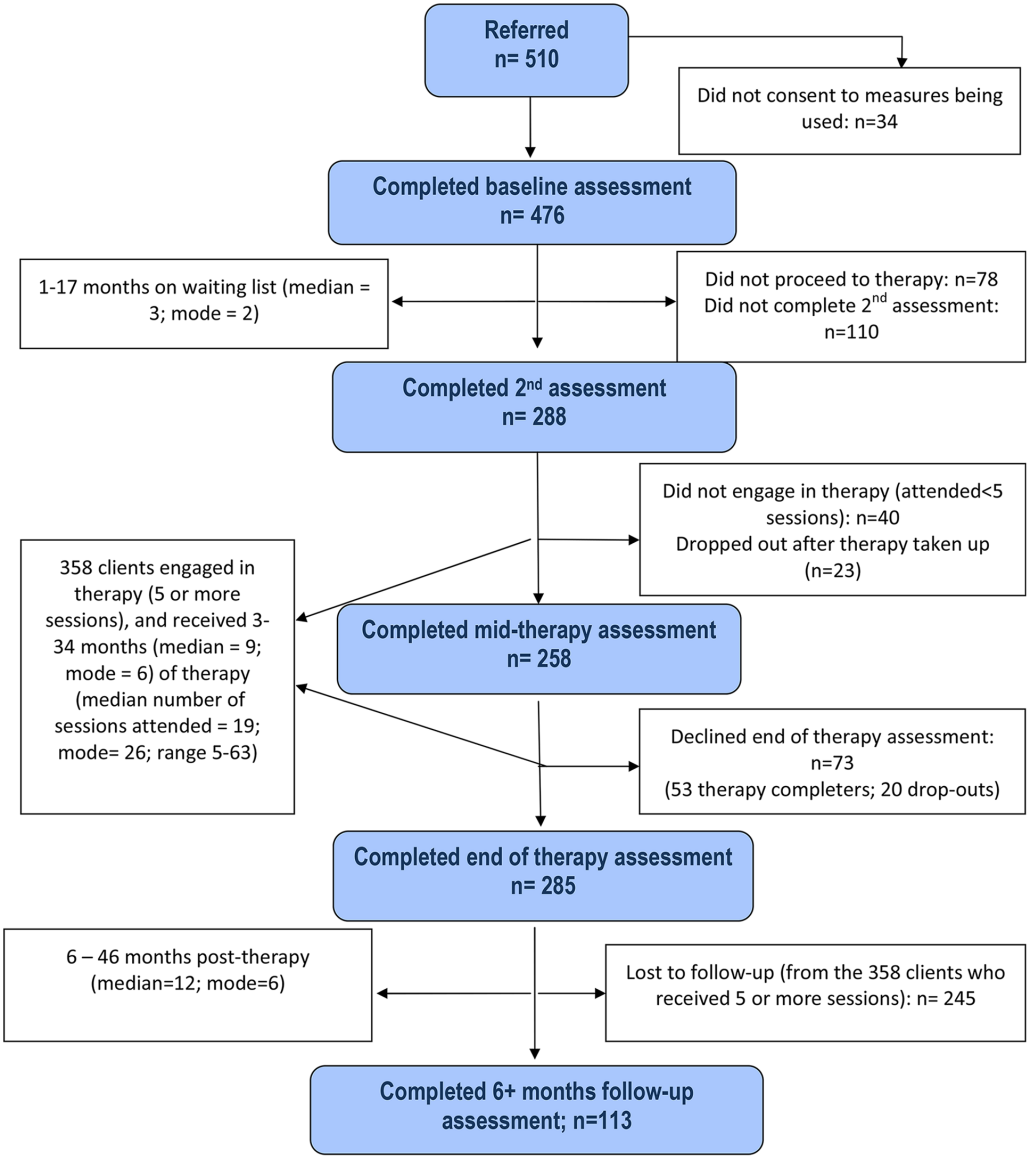
**Consort diagram of closed cases**.

**FIGURE 2:**
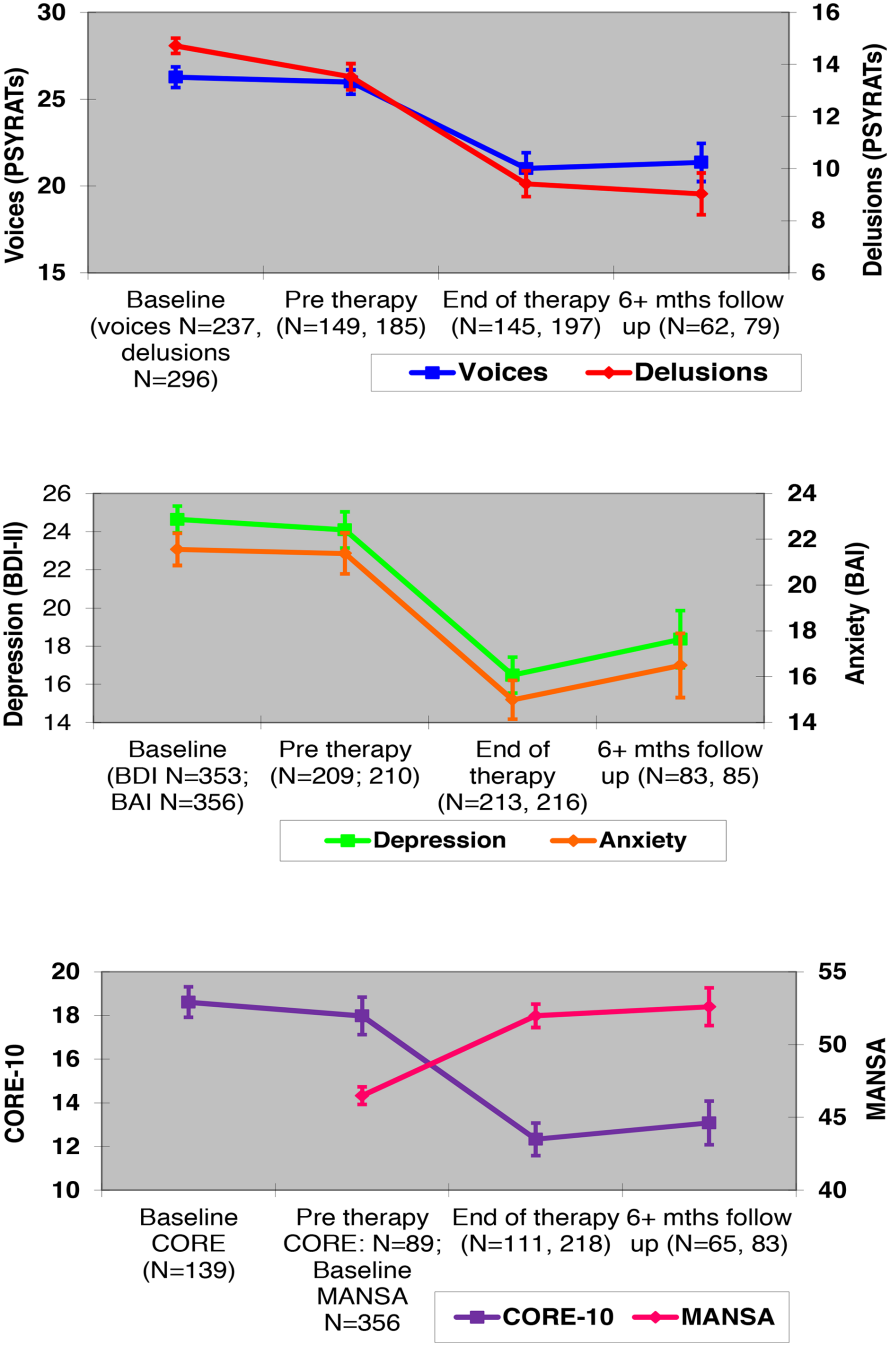
**Means (with standard errors) of clinical outcomes at each assessment time-point.** PSYRATs, Psychotic Symptom Rating Scales (Haddock et al., 1999). BDI-II and BAI, Beck Depression Inventory-II (Beck et al., 1996) and Beck Anxiety Inventory (Beck and Steer, 1990). CORE-10, Clinical Outcomes in Routine Evaluation-10 (Barkham et al., 2013). MANSA, Manchester Short Assessment of Quality of Life Questionnaire (Priebe et al., 1999).

Attrition rates at each stage of assessment and therapy drop-outs are illustrated in the service consort diagram (see **Figure 1**). Clinical scores at each assessment stage (apart from mid-therapy) on each of the six outcome measures are shown in **Figure 2.**

Of the 476 consenting cases, a further 118 people were excluded from further assessments because they either did not proceed to therapy [*N* = 78 (16%)], or they dropped out of therapy too early to receive a meaningful ‘dose’ [defined ‘*a priori*’ as attending fewer than five sessions^4^; *N* = 40 (8%)], according to the clinic’s procedures. They did not differ significantly from the 358 people who engaged in therapy (five or more sessions attended) on gender (χ^2^ = 0.04, d.f. = 1, *p* > 0.1), age (*t* = 1.6, d.f. = 473, *p* = 0.11), ethnicity (χ^2^ = 0.15, d.f. = 1, *p* > 0.1), or marital status (χ^2^ = 3.07, d.f. = 3, *p* > 0.1), or on any of the baseline clinical variables [PSYRATS–H (Haddock et al., 1999): *t* = 0.11, d.f. = 235, *p* > 0.1; PSYRATS–D (Haddock et al., 1999): *t* = 1.65, d.f. = 294, *p* = 0.10; BDI-II (Beck et al., 1996): *t* = 2.25, d.f. = 351, *p* = 0.03; BAI (Beck et al., 1988): *t* = 1.98, d.f. = 354, *p* = 0.05; CORE-10 (Barkham et al., 2013): *t* = 0.68, d.f. = 137, *p* > 0.1, and MANSA (Priebe et al., 1999): *t* = 2.04, d.f. = 354, *p* = 0.05].

Of those who started therapy (398 people), 110 (28%) did not complete a second assessment (either because they declined or there was a therapist available within 2 weeks of baseline assessment).

Of those who initially engaged in therapy (attended five or more sessions; 358 people), 23 (5%) dropped out later on in therapy, giving a total drop-out rate of 13% [i.e., including those who did not engage (*N* = 40; 8%), and those who took up therapy but later dropped out].

Of the 358 people who engaged in therapy, 73 (20%; 53 therapy completers and 20 drop-outs) declined an end of therapy assessment, although 56% of them agreed to a mid-therapy assessment (*N* = 36; 31 therapy completers and five drop-outs) and/or a follow-up *(N* = 5; five therapy completers and zero drop-out) assessment. Those who declined the end of therapy assessment did not differ significantly from the 285 people who completed it on gender (χ^2^ = 0.87, d.f. = 1, *p* > 0.1), age (*t* = 1.5, d.f. = 355, *p* > 0.1), ethnicity (χ^2^ = 0.14, d.f. = 1, *p* > 0.1), or marital status [χ^2^ = 5.7, d.f. = 3, *p* > 0.1)], or on any of the baseline clinical variables [PSYRATS–H (Haddock et al., 1999): *t* = 0.96, d.f. = 178, *p* > 0.1; PSYRATS–D (Haddock et al., 1999): *t* = 1.38, d.f. = 237, *p* > 0.1; BDI-II (Beck et al., 1996): *t* = 0.41, d.f. = 271, *p* > 0.1; BAI (Beck et al., 1988): *t* = 2.02, d.f. = 272, *p* = 0.05, CORE-10 (Barkham et al., 2013): *t* = 1.23, d.f. = 105, *p* > 0.1, or MANSA (Priebe et al., 1999): *t* = 2.31, d.f. = 281, *p* = 0.02].

A significant number (*N* = 245; 68% of those who attended five or more sessions) were lost to follow-up (see procedures section). The 113 individuals who completed a 6+ months follow-up assessment did not differ from those who did not on gender (χ^2^ = 3.0, d.f. = 1, *p* = 0.08), age (*t* = 0.59, d.f. = 355, *p* > 0.1), ethnicity (χ^2^ = 0.4, d.f. = 1, *p* > 0.1), or marital status (χ^2^ = 7.6, d.f. = 3, *p* = 0.06), or on any of the baseline clinical variables [PSYRATS–H (Haddock et al., 1999): *t* = 0.10, d.f. = 178, *p* > 0.1; PSYRATS–D (Haddock et al., 1999): *t* = 9.2, d.f. = 237, *p* > 0.1;[BDI-II (Beck et al., 1996): *t* = 0.55, d.f. = 271, *p* > 0.1; BAI (Beck et al., 1988): *t* = 1.4, d.f. = 272, *p* > 0.1), CORE-10 (Barkham et al., 2013): *t* = 0.6, d.f. = 105, *p* > 0.1, or MANSA (Priebe et al., 1999): *t* = 0.04, d.f. = 281, *p* > 0.1].

Furthermore, those who did not complete a follow-up assessment did not differ significantly from those who did at the end of therapy (or mid-therapy for those who did not complete an end of therapy assessment) on any of the clinical variables [PSYRATS–H (Haddock et al., 1999): *t* = 0.78, d.f. = 164, *p* > 0.1; PSYRATS–D (Haddock et al., 1999): *t* = 0.34, d.f. = 219, *p* > 0.1; BDI-II (Beck et al., 1996): *t* = 1.07, d.f. = 244, *p* > 0.1; BAI (Beck et al., 1988): *t* = 1.26, d.f. = 245, *p* > 0.1, CORE-10 (Barkham et al., 2013): *t* = 1.72, d.f. = 118, *p* = 0.09, or MANSA (Priebe et al., 1999): *t* = 0.71, d.f. = 216, *p* > 0.1].

**Table 1:** Mixed Effects Regression Results for the Effectiveness of CBTp.

Variable/Contrast	Coefficient	*SE*	*P*-value	Effect size
**PSYRATS voices – Total (248 individuals)**				
Baseline vs. pre-therapy	-0.46	0.57	0.42	0.05
Pre-therapy vs. post-therapy	-4.65	0.83	**<0.001**	**0.52**
Change baseline/pre-therapy vs. change pre/post-therapy^∗^	-4.18	1.20	**<0.001**	
Pre-therapy vs. follow-up	-3.89	1.05	**<0.001**	**0.44**
Post-therapy vs. follow-up	0.76	1.04	0.47	0.09
**PSYRATS delusions – Total (302 individuals)**				
Baseline vs. pre-therapy	-1.23	0.42	** 0.003**	**0.23**
Pre-therapy vs. post-therapy	-3.99	0.49	**<0.001**	**0.75**
Change baseline/pre-therapy vs. change pre/post-therapy	-2.75	0.77	**<0.001**	
Pre-therapy vs. follow-up	-4.34	0.76	**<0.001**	**0.82**
Post-therapy vs. follow-up	0.35	0.70	0.61	0.07
**BDI (360 individuals)**				
Baseline vs. pre-therapy	-1.45	0.59	0.014	0.11
Pre-therapy vs. post-therapy	-6.75	0.75	**<0.001**	**0.51**
Change baseline/pre-therapy vs. change pre/post-therapy	-5.30	1.15	**<0.001**	
Pre-therapy vs. follow-up	-4.44	1.04	**<0.001**	**0.34**
Post-therapy vs. follow-up	2.31	0.91	** 0.01**	**0.18**
**BAI (362 individuals)**				
Baseline vs. pre-therapy	-0.78	0.65	0.23	0.06
Pre-therapy vs. post-therapy	-5.66	0.81	**<0.001**	**0.44**
Change baseline/pre-therapy vs. change pre/post-therapy	-4.87	1.26	**<0.001**	
Pre-therapy vs. follow-up	-3.73	1.01	**<0.001**	**0.29**
Post-therapy vs. follow-up	1.93	0.95	0.04	0.15
**CORE -Total (180 individuals)**				
Baseline vs. pre-therapy	-1.42	0.67	0.03	0.17
Pre-therapy vs. post-therapy	-5.18	0.72	**<0.001**	**0.61**
Change baseline/pre-therapy vs. change pre/post-therapy	-3.76	1.20	**<0.002**	
Pre-therapy vs. follow-up	-3.95	0.90	**<0.001**	**0.47**
Post-therapy vs. follow-up	1.23	0.71	0.08	0.15
**MANSA (361 individuals)**				
Pre-therapy vs. post-therapy	5.30	0.69	**<0.001**	**0.49**
Pre-therapy vs. follow-up	5.01	1.06	**<0.001**	**0.47**
Post-therapy vs. follow-up	-0.29	0.92	0.75	0.03


*^∗^Is the change between pre vs. post-therapy significantly greater than the change between baseline vs. second assessment?*

*Significant results (p < 0.01) in bold.*

### Outcome Analyses

The residuals from the six different linear models were approximately normally distributed, denoting that the model assumptions are plausible.

Results are displayed in **Table 1.** Total numbers available for the six mixed-effects regressions for each outcome were as follows: PSYRATS-Voices = 248; PSYRATS-Delusions = 302; BDI = 360; BAI = 362; CORE = 180; MANSA = 361. Results are provided for the following contrasts: baseline vs. pre-therapy (i.e., changes during the waiting list); pre- vs. post-therapy (i.e., changes during the therapy); pre-therapy vs. follow-up (i.e., changes during therapy + follow-up period); post-therapy vs. follow-up (i.e., changes between the end of therapy and follow-up). Finally the comparison between amount of change during therapy and amount of change during waiting list is also reported.

It can be seen that clients’ symptoms remained stable during the waiting list period, with all comparisons^5^ being either non-significant (voices; depression; anxiety; and well-being) or with low effect sizes (delusions; <=0.23). In contrast, all outcomes improved significantly after therapy (pre vs. post; all *p* < 0.001), and were maintained at the follow-up stage (pre vs. follow-up, also all *p* < 0.001), with effect sizes ranging from 0.44 to 0.75 at the end of therapy, and 0.29 to 0.82 at follow-up. Overall the effect sizes for both comparisons were largest for delusions, and smallest for anxiety. The change during therapy (post therapy – pre-therapy) was significantly greater than that occurring during the waiting list (pre therapy – baseline) on all available measures. There was little change between end of therapy and follow-up, with all comparisons either being non-significant (voices; delusions; anxiety; well-being; qualify of life) or with low effect size (depression; <=0.18), indicating that clients did not deteriorate following the end of therapy, although they did not continue to improve either.

## Discussion

In one of the largest effectiveness study of its kind in psychosis, we provide evidence for the long term effectiveness of CBTp on a range of meaningful outcomes, delivered in a UK, NHS psychological therapies service. Bearing in mind that this study reported on a consecutive sample with a wide range of presentations from an ethnically diverse, socially deprived and high mobility area; that drop-out rate from therapy was low (13%); and that patients were seen by therapists with a wide range of experience in CBTp, these results are encouraging. They add support to the evidence-base from RCTs that suggests that people who have ongoing, residual distressing symptoms of psychosis and emotional difficulties represent one of the groups most likely to benefit from CBTp (Burns et al., 2014). They confirm that psychological well-being, emotional difficulties and quality of life can also be improved by psychological therapy, in addition to symptom-associated distress and disability.

### Strengths

The results should be interpreted within the context of a number of strengths and limitations. One of the strengths was the large sample size, obtained from consecutive referrals over a 12 years period. The sample was representative of the heterogeneity and complexity of individuals presenting with psychotic symptoms, unlike RCTs that have been criticized on the basis of ‘cherry-picking’ their participants. As a service the PICuP clinic has an inclusive suitability policy: referrals are deemed appropriate as long as people are presenting with distressing symptoms of psychosis and/or emotional difficulties in the context of a history of psychosis, and are willing and able to attend sessions. We accept referrals with any diagnosis (or indeed diagnostic conundrums), any type and severity of symptomatic presentation, including co-morbidities, any type of medication (or no medication), any level of cognitive ability, and any model of understanding of psychotic experiences, i.e., having clinical insight is not a pre-requisite. The only exclusion criteria are a primary diagnosis of substance abuse (such as hallucinations caused entirely by alcohol abuse), and a current, very high risk of harming others. People who have a dual diagnosis with substance dependence, or are at risk of self-harm, are accepted by the service. In practice, our service-users tend to be those who do not need an assertive outreach service, i.e., they do not have substantial negative symptoms and/or have predominantly unmet social needs, and do not show severe chaotic behavior that would prevent them from being able to attend any sessions.

Another important strength was the large number of therapists (*N* = 121), most of whom were not expert in CBTp, and included clinical psychologists in training. The relatively large and enduring effects show that this type of therapy can be successfully implemented in an NHS setting with therapists with a range of experience. However, similarly to our trial (Peters et al., 2010), four crucial aspects of the therapy delivery were likely to have facilitated good outcomes (see also Jolley et al., 2015). First, all therapists had received training in CBT already, and most had a doctoral qualification (e.g., Doctorate in Clinical Psychology), ensuring they were already familiar with the cognitive model and general concepts of CBT, and had some basic understanding of psychological approaches to psychosis. Second, the service has a well-established supervision structure, ensuring they all received regular clinical supervision by senior staff specializing in CBTp (fortnightly in a group setting for qualified staff, and individual weekly supervision for trainees).

Third, PICuP is a stand-alone psychological therapies service that operates independently from the referring teams, although therapists liaise closely with the referrer about the progress of their individual clients. This service context meant that therapists had assured protected time for the delivery of the therapy and attendance at supervision, free from competing demands of multidisciplinary team work, whether as permanent staff in the PICuP clinic or as CPD therapists employed in another setting. There is increasing evidence that attempts to deliver complex therapies such as CBTp by care-coordinators or staff with limited training, or by adequately trained therapists but without protected time or supervision, are not likely to be productive (Brooker and Brabban, 2004; Brosan et al., 2006; Steel et al., 2012). Finally, the specialized nature of the service ensured both an awareness of how to accommodate the difficulties facing people with psychosis by all staff, including assessors, as well as a predominant culture embracing a psychological approach to psychosis (Cooke, 2014).

Further strengths included the use of independent assessors, rather than outcomes being elicited by the therapists themselves, and the availability of data from mid-therapy assessments for five out of the six measures. The inclusion of mid-therapy outcomes in the analyses meant that potential bias created by missing values at the end of therapy assessment was reduced. Lastly the follow-up period was of reasonable length (median of 12 months post therapy), with the maximum being 46 months after having finished therapy.

The PICuP service was set-up as part of a funded RCT (Peters et al., 2010), and therefore its model of therapy delivery and outcomes monitoring mirrored closely the high standards of RCTs, which can be difficult to achieve in routine community services. However, it has been demonstrated recently (Jolley et al., 2015) that this service model can be implemented on a larger scale across different pathways of care [achieved with additional funding from NHS England for the Improving Access to Psychological Therapies for Severe Mental Illness (IAPT-SMI) initiative], with the important variables being the employment of appropriately trained therapists, access to regular supervision, protected time to deliver therapy, and the use of independent assessors. Whether this service model can be implemented in different health service contexts across countries remains to be investigated.

### Limitations

The study also had limitations. The reported effects are within participants only, with no untreated or control therapy group, and the results therefore cannot be unambiguously interpreted as being due to the therapy. However, a number of factors suggest that the reported benefits are unlikely to represent natural recovery. First, our sample consisted largely of a fairly stable group with residual symptoms, rather than an early intervention or frequently relapsing group; in our trial the median length of illness was found to be 6.5 years (Peters et al., 2010). Second, no meaningful changes were found on any of the measures used while patients were on the waiting list, apart from a slight decrease in delusions (effect size = 0.23). Importantly, the differences in outcomes between pre- and post-therapy assessments were significantly greater than those between baseline and pre-therapy for all outcomes where this was available. It is also unlikely that the results are due to natural fluctuations in symptoms, since outcomes remained stable both before and following therapy, with the latter period being greater (median of 12 months) than the length of therapy (median of 9 months).

The assessments were conducted by independent psychology assistants, but they were not blind to the specific assessment time-point, meaning that effects may have been inflated by the expectations of the assessors. However, four of the six measures evaluated consisted of self-report, and would therefore not have been subject to assessor bias; their effect sizes were broadly equivalent to those obtained from interviewer-rated measures.

A third limitation was that we had limited assessments on those who dropped-out of therapy. Due to resource constraints on the clinic, it was decided a-priori that those who did not engage in therapy (i.e., attended fewer than five sessions) would not be pursued for further assessments. Although it was attempted to follow up those who engaged, but dropped out of therapy at a later stage (i.e., attended five sessions or more), only a minority agreed to be assessed (13%), although a further 20% had mid-therapy data available. Nevertheless, once therapy was started the number of drop-outs was low overall (13%: 8% did not engage, and 5% dropped out at a later stage), and therefore it is unlikely to have created a significant bias in the overall findings.

Although the overall sample size was large (number of cases available for analyses ranged from 180 to 362, depending on outcome), there were large amounts of missing data on some scales, due to their intermittent use throughout the 12 years of the service (due to financial constraints or NHS Trust initiatives). There was also a sizeable proportion (28%) who did not have waiting list data due to missing assessments or immediate allocation of a therapist.

Perhaps the most important limitation was that the follow-up assessments were conducted on only a sub-set of the sample who engaged in therapy (32%). This was partly because they were only implemented as a routine procedure 7 years after the start of the service, and partly because they tend to be de-prioritized in a busy clinical setting. Although those who were followed-up did not differ on any demographic or clinical variable, either at baseline or at the end of therapy, it remains unclear whether loss to follow-up was random. It is possible that those who feel they benefited from therapy may be more willing to agree to attend a follow-up assessment than others, thus creating a possible bias toward an overestimate of treatment effects at longer term follow-up. On the other hand, it is also possible that some people who are not doing well may be motivated to come back for an assessment in order to access booster sessions (six booster sessions are available to all those who request it). It is clearly desirable to obtain a much higher follow-up rate, although this is a difficult task to achieve in the context of routine clinical services. Overall it cannot be assumed that the long-term outcomes found would generalize to the rest of the sample, and the findings therefore have to be interpreted with this important caveat in mind.

Other limitations included the lack of data available on medication changes during therapy (or indeed any of the other periods assessed), although in general this has not been found to be a moderating factor in CBTp RCTs. Our sample may not have been representative of all outpatients with psychosis; as a psychological therapies service we are dependent on referrals from other professionals (although a minority of our patients also self-refer), and we tend not to see people with both socially complex and chaotic presentations, who are better seen by therapists working within multidisciplinary teams. This means that our clients tend to be motivated to attend therapy, as is illustrated by the low drop-out rate.

## Conclusion

This study has important implications for the practice of CBTp. It demonstrates that CBTp can have a positive impact on clients’ experience of positive symptoms, levels of depression and anxiety and overall well-being and satisfaction with their life, even when conducted in a routine psychological therapies service by CBT therapists with a range of experience in psychosis, as long as people have regular supervision and protected time. It also provides promising evidence that gains can be maintained long-term, and opens the door for further research to explore which aspects of CBTp have the most impact long-term, and how we can aid the maintenance of therapy gains.

## Conflict of Interest Statement

The authors declare that the research was conducted in the absence of any commercial or financial relationships that could be construed as a potential conflict of interest.

## Footnotes

1 There was one participant with missing data for age; 47 people did not wish to disclose their ethnicity, and 29 did not reveal their marital status. There were 6 people with missing data on the PSYRATS-H, 4 on the PSYRATS-D, 123 on the BDI, and 120 on the BAI (see section on measures for an explanation of the large amount of missing data on BDI and BAI).

2 Data on therapy duration were obtained from assessment dates; a few patients were put on hold during the course of therapy, explaining the upper ranges of therapy duration.

3 1% rather than 5% significance level was used for all analyses to account for multiple testing.

4 <5 sessions was based on CBTp guidance that the first 4–6 sessions are usually devoted to engagement only.

5 Quality of life (i.e., MANSA) data were not available for this comparison.
